# Differentiation between multiple sclerosis and neuromyelitis optica spectrum disorder using optical coherence tomography angiography

**DOI:** 10.1038/s41598-021-90036-6

**Published:** 2021-05-21

**Authors:** Małgorzata Rogaczewska, Sławomir Michalak, Marcin Stopa

**Affiliations:** 1 Department of Ophthalmology, Chair of Ophthalmology and Optometry, Poznan University of Medical Sciences, ul. Grunwaldzka 16/18, 60-780 Poznan, Poland; 2 Department of Neurochemistry and Neuropathology, Chair of Neurology, Poznan University of Medical Sciences, ul. Przybyszewskiego 49, 60-355 Poznan, Poland

**Keywords:** Multiple sclerosis, Optic nerve diseases

## Abstract

Neuromyelitis optica spectrum disorder (NMOSD) and multiple sclerosis (MS) are autoimmune demyelinating diseases of distinct etiology presenting with optic neuritis (ON). This study aimed to identify the macular and peripapillary neurovascular alterations that may facilitate the differentiation between NMOSD and MS eyes using spectral-domain optical coherence tomography (OCT) and OCT angiography (OCTA). A total of 13 NMOSD patients and 40 MS patients were evaluated. After ON, the radial peripapillary capillary (RPC) vessel density was significantly decreased in the superior (S) and inferior (I) sectors in NMOSD compared with MS eyes, whereas in non-ON eyes, the temporal (T) sector of RPC was reduced in MS group. In the ON eyes, the retinal nerve fiber layer in the I and T quadrants was thinner in NMOSD than in MS. Regarding ON and non-ON eyes, the macular capillary plexuses, and the ganglion cell complex thickness did not differ between NMOSD and MS. The ratios, based on the disease-specific intra-eye RPC vessel density reduction pattern, were the best discriminants between NMOSD and MS, i.e., inferior to nasal (I/N) and I/T ratios for ON eyes, and S/T and N/T ratios for non-ON eyes. Our results show that the OCTA-based simple ratios may be useful in distinguishing NMOSD and MS patients.

## Introduction

Neuromyelitis optica spectrum disorder (NMOSD) and multiple sclerosis (MS) are autoimmune demyelinating diseases affecting the central nervous system that may present with optic neuritis (ON)^1,2^. Although the clinical manifestation of NMOSD may resemble MS, the NMOSD-specific serum autoantibody can be detected in up to 80% of patients, confirming distinct etiology of the disease^3,4^.

The retinal architecture can be evaluated non-invasively in vivo using spectral-domain optical coherence tomography (SD-OCT) for ganglion cell complex (GCC) and retinal nerve fiber layer (RNFL) thickness quantification, and OCT angiography (OCTA) for macular and peripapillary vessel density assessment^5,6^.

Several studies showed that after ON, the GCC and RNFL thickness were more reduced in NMOSD eyes than in MS eyes^7–10^. Comparing NMOSD and MS eyes, Green and Cree found the attenuation of the peripapillary vessels on fundoscopic examination that characterized the NMOSD eyes^11^. Recently Lee et al. reported that in ON eyes, the superficial macular and radial peripapillary capillary (RPC) plexus vessel density were lower in NMOSD than MS eyes^10^. However, a similar OCTA study of the non-ON eyes has not been reported so far.

As the etiology of NMOSD and MS differs, the comparative analysis of OCT measurements may indicate the characteristic pattern of lesions in ON and non-ON eyes differentiating these diseases. Moreover, the ratio based on the potential disease-specific intra-eye changes may also be a possible discriminant between NMOSD and MS eyes.

In the current study, we aimed to identify the macular and peripapillary neurovascular alterations through SD-OCT and OCTA to differentiate NMOSD from MS eyes.

## Methods

### Study participants

In this single-center study, we recruited MS and NMOSD patients from the Department of Ophthalmology and the Department of Neurology of the Poznan University of Medical Sciences between June 2018 and September 2020. All 40 MS patients had a relapsing–remitting disease and fulfilled the revised 2017 McDonald criteria^12^. NMOSD patients were diagnosed according to the revised 2015 NMOSD diagnostic criteria^13^. The anti-aquaporin-4 antibodies (AQP4-IgG) were detected employing indirect fluorescence using a commercial cell-based assay with aquaporin 4 transfected cells (EUROIMMUN AG, Lübeck, Germany). In total, 24 patients were diagnosed with AQP4-IgG seropositive NMOSD in our institution. Seven patients could not undergo ophthalmic examination because of visual or physical disability, and four patients refused to participate. Thus, only 13 patients were enrolled in this study. Clinical data, including the history of ON and disease duration, were obtained through chart review. The disease-modifying therapy for MS patients was: glatiramer acetate (n = 8), dimethyl fumarate (n = 8), teriflunomide (n = 1), interferon beta-1a (n = 5), interferon beta-1b (n = 7), fingolimod (n = 10), natalizumab (n = 1). The NMOSD patients were receiving azathioprine (n = 5) and intermittent intravenous immunoglobulin (n = 1), while the other patients were not treated at the time of study enrollment.

The eyes of MS and NMOSD patients were classified as eyes with a history of ON (MS + ON, NMOSD + ON), and without the history of ON (MS-ON, NMOSD-ON).

Each patient underwent slit-lamp biomicroscopy, fundus examination, Goldmann applanation tonometry with central corneal thickness correction, SD-OCT, and OCTA. Best-corrected visual acuity (BCVA) was assessed with the Early Treatment of Diabetic Retinopathy Study (ETDRS) chart and expressed as a logarithm of the minimum angle of resolution (logMAR).

Eligibility criteria were age ≥ 18 years, no ON attack within the last 6 months before the examination, and at least 2 years of disease duration for MS patients. Exclusion criteria were myopia > 6 diopters, glaucoma, optic disc drusen, macular disease, hypertensive or diabetic retinopathy, history of uveitis or eye surgery, and low OCT image quality.

The research was performed in accordance with the Declaration of Helsinki and was approved by the medical ethics committee of Poznan University of Medical Sciences (approval No. 562/18 from May 2018). Written informed consent was obtained from all patients after a full explanation of the study.

### SD-OCT

The ganglion cell complex and peripapillary retinal nerve fiber layer thickness were obtained with RTVue XR Avanti with AngioVue (Optovue Inc., Fremont, CA, USA; software version 2017.1.0.151). The GCC scan, covering a square grid (7 mm × 7 mm) on the macula, was centered 1 mm temporal to the fovea. The device automatically measured the average GCC thickness from the internal limiting membrane (ILM) to the outer boundary of the inner plexiform layer (IPL). The RNFL thickness was acquired using the optic nerve head map protocol and measured at a diameter of 3.45 mm around the center of the optic disc. The average RNFL thickness and the thickness in each quadrant, i.e., superior (S), nasal (N), inferior (I), and temporal (T) were analyzed. The OSCAR-IB criteria were used for quality control of OCT images^14^.

### OCT angiography

The OCTA image acquisition was performed with RTVue XR Avanti with AngioVue (Optovue Inc., Fremont, CA, USA; software version 2017.1.0.151), which is based on a split-spectrum amplitude-decorrelation angiography algorithm. This non-invasive imaging modality detects the motion of erythrocytes in the vessels through sequentially obtained OCT cross-sectional scans. The blood flow maps present the vessel density, defined as the percentage area occupied by the perfused retinal blood vessels in the analyzed region^15,16^.

The parafoveal vessel density was visualized using a 3 × 3 mm scan centered on the fovea. The AngioVue software segmented the 3-dimensional image of the retinal capillaries into two plexuses, i.e., the superficial capillary plexus (SCP; from ILM to the 9 μm above the IPL) and the deep capillary plexus (DCP; from 9 μm above the IPL to the 9 μm below the outer plexiform layer). The parafoveal area was defined as an annulus with an inner diameter of 1.0 mm and an outer diameter of 3.0 mm. Furthermore, the annulus was automatically divided into quadrants (S, N, I, T), according to the ETDRS grid^17^. We analyzed the average and sectoral vessel density of SCP and DCP. Additionally, the software provided the sectoral thickness of parafoveal GCC, which was also evaluated.

To visualize the RPC plexus, a 4.5 × 4.5 mm rectangle scan centered on the optic nerve head was used, and the peripapillary region was defined as a 1.0-mm wide round annulus extending outward from the optic disc boundary. The capillaries within the ILM and the outer boundary of the nerve fiber layer were automatically analyzed. We assessed the average and quadrant RPC vessel density.

The low-quality OCTA images, i.e., with the signal strength index < 50, scan quality < 7/10 or motion artifacts, were rejected. The OCT data were reported following APOSTEL recommendations^18^.

### Statistical analysis

Statistical analysis was performed using Statistica v13.3 (StatSoft, Inc., Tulsa, USA) and SPSS (SPSS, Inc., Chicago, USA). The Shapiro–Wilk test was used to determine the distribution of the data. Differences between the groups were tested using a Chi-square test for sex, an unpaired Student *t*-test for age, and a Mann–Whitney *U*-test for disease duration, number of ON attacks, and BCVA. We performed generalized estimating equation models accounting for within-patient inter-eye dependencies to compare the SD-OCT and OCTA measurements between cohorts. The area under the receiver operating characteristic (ROC) curve (AUC) was used to calculate the diagnostic power of the selected parameters in differentiating between NMOSD and MS eyes. Statistical significance was established at *p* < 0.05.

## Results

### Study population

The clinical characteristics of the NMOSD and MS patients are detailed in Table 1. The groups were matched for sex and disease duration and differed in age (*p* = 0.011). The BCVA of enrolled eyes was worse in NMOSD than MS for ON and non-ON eyes (*p* = 0.013 and *p* = 0.045, respectively). The number of ON attacks did not differ between NMOSD and MS (*p* = 0.352). We excluded 3 eyes of MS and 6 eyes of NMOSD patients from analysis because of the low-quality OCTA images.

**Table 1:** Demographic and clinical summary of patients with NMOSD and MS.

	NMOSD	MS
Number of patients	13	40
Sex (female/male)	11/2	32/8
Age (years, mean ± SD)	42.08 ± 10.23	35.15 ± 7.47
Disease duration (years, median, range)	9 (1–33)	8 (3–32)
Number of eyes enrolled	20	77
ON eyes	9	31
Non-ON eyes	11	46
No. of ON attacks (median, range)	1 (1–3)	1 (1–3)
Bilateral ON, *n* (%)	5 (38.5)	6 (15.0)
Unilateral ON, *n* (%)	5 (38.5)	20 (50.0)
No history of ON, *n* (%)	3 (23.0)	14 (35.0)
BCVA of enrolled eyes (logMAR, median, range)		
ON eyes	0.00 (0.00–2.30)	0.00 (0.00–0.20)
Non-ON eyes	0.00 (0.00–0.10)	0.00 (0.00–0.00)

*BCVA* Best-corrected visual acuity, *logMAR* The logarithm of the minimum angle of resolution, *MS* Multiple sclerosis, *NMOSD* Neuromyelitis optica spectrum disorder, *ON* Optic neuritis, *SD* Standard deviation.

### Comparison of SD-OCT and OCTA measurements in ON eyes

**Figure 1:**
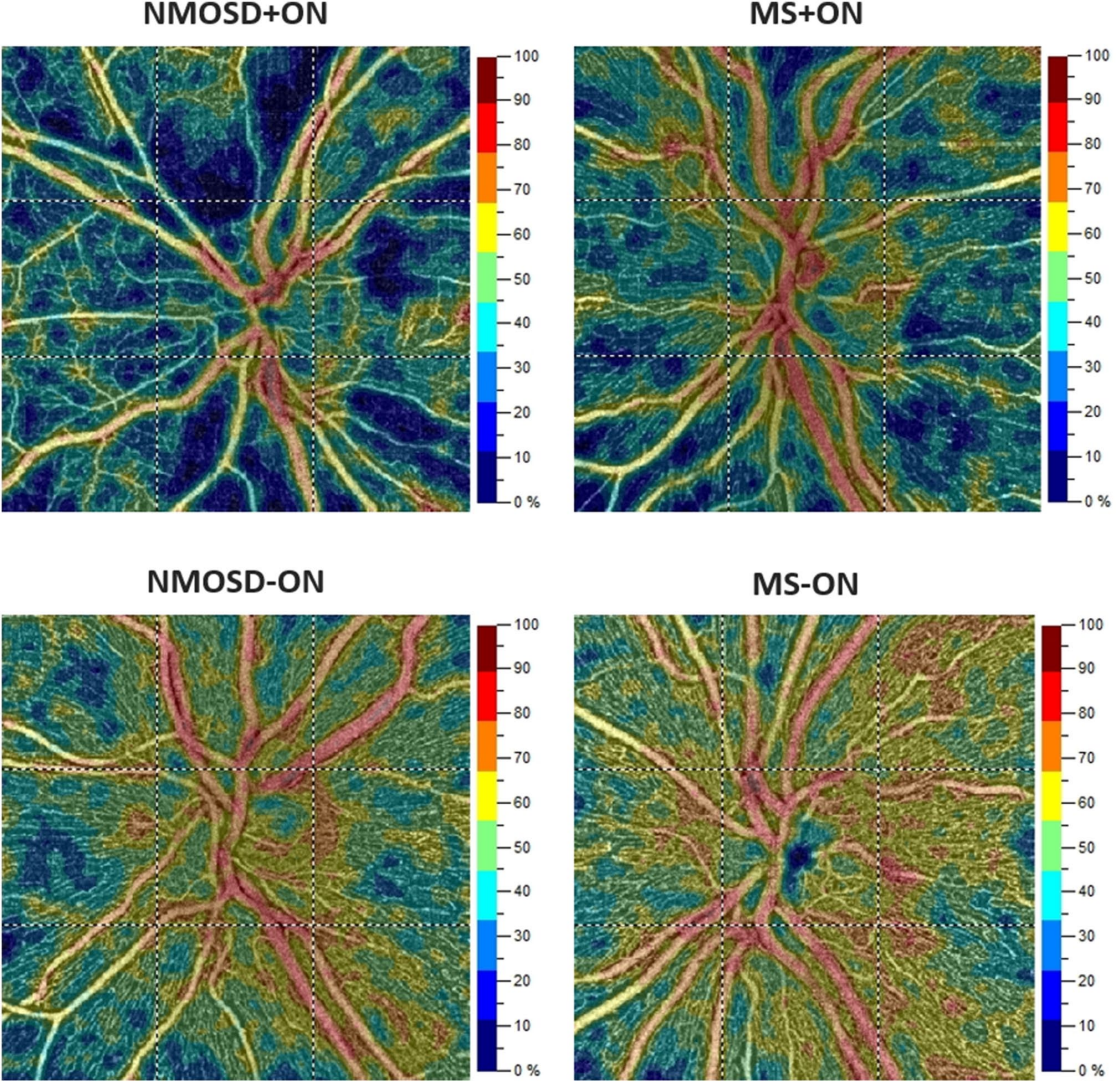
Exemplary OCT angiography images of the NMOSD + ON, MS + ON, NMOSD-ON, and MS-ON left eyes. The color-coded flow density maps of the peripapillary capillary plexus present reduced vessel density in eyes with a history of optic neuritis, more marked in NMOSD + ON eye. The narrowing of retinal vessels is also seen in NMOSD + ON eye. *MS* Multiple sclerosis, *NMOSD* Neuromyelitis optica spectrum disorder, *OCT* Optical coherence tomography, *ON* Optic neuritis.

The average and sectoral vessel density of SCP and DCP, as well as the GCC thickness, did not differ between NMOSD and MS (Table 2). In NMOSD + ON eyes, the RPC vessel density was reduced in the superior (*p* = 0.040) and inferior (*p* = 0.019) quadrants compared with MS + ON eyes (Table 2, Fig. 1). The created with these quadrants ratios, i.e., superior to temporal (S/T), inferior to nasal (I/N), and inferior to temporal (I/T), were significantly lower in NMOSD + ON eyes (Table 2). The RNFL was thinner in NMOSD + ON eyes in the inferior (*p* = 0.043) and temporal (*p* = 0.047) quadrants. However, the RNFL quadrant-based ratios did not differ between the NMOSD and MS patients (Table 2).

**Table 2:** Comparison of ON eyes between NMOSD and MS patients.

	NMOSD + ON	MS + ON	NMOSD + ON versus MS + ON		
SCP (%)	Mean ± SD	Mean ± SD	β	95% CI	*p* value
Average	42.43 ± 7.00	43.60 ± 4.47	− 1.175	(− 6.043 to 3.693)	0.636
Superior	43.44 ± 7.53	44.30 ± 5.08	− 0.863	(− 6.379 to 4.654)	0.759
Nasal	40.98 ± 8.06	42.85 ± 5.05	− 1.877	(− 7.411 to 3.658)	0.506
Inferior	43.69 ± 6.86	44.82 ± 4.82	− 1.129	(− 5.855 to 3.598)	0.640
Temporal	41.60 ± 6.03	42.38 ± 3.66	− 0.784	(− 4.878 to 3.310)	0.707
**DCP (%)**					
Average	55.91 ± 2.29	56.97 ± 2.20	− 1.062	(− 2.684 to 0.560)	0.200
Superior	55.29 ± 2.42	56.97 ± 2.73	− 1.680	(− 3.629 to 0.269)	0.091
Nasal	55.66 ± 2.79	56.90 ± 2.55	− 1.234	(− 3.164 to 0.696)	0.210
Inferior	56.68 ± 2.72	57.23 ± 2.66	− 0.554	(− 2.382 to 1.274)	0.552
Temporal	56.00 ± 2.43	56.80 ± 1.92	− 0.800	(− 2.434 to 0.834)	0.337
**RPC (%)**					
Average	40.80 ± 8.99	46.76 ± 4.96	− 5.963	(− 12.556 to 0.629)	0.076
Superior	40.17 ± 11.07	48.68 ± 6.34	− 8.493	(− 16.583 to − 0.403)	0.040
Nasal	39.70 ± 8.09	44.53 ± 5.55	− 4.817	(− 10.963 to 1.328)	0.124
Inferior	41.27 ± 11.38	51.20 ± 5.97	− 9.913	(− 18.188 to − 1.638)	0.019
Temporal	42.66 ± 6.86	44.06 ± 6.89	− 1.386	(− 7.112 to 4.341)	0.635
S/N ratio	0.99 ± 0.14	1.10 ± 0.12	− 0.099	(− 0.207 to 0.010)	0.074
S/T ratio	0.93 ± 0.16	1.12 ± 0.20	− 0.196	(− 0.335 to − 0.057)	0.006
I/N ratio	1.02 ± 0.12	1.16 ± 0.10	− 0.131	(− 0.221 to − 0.041)	0.004
I/T ratio	0.95 ± 0.15	1.18 ± 0.19	− 0.230	(− 0.357 to − 0.103)	< 0.001
**GCC (μm)**					
Average	77.75 ± 10.70	83.61 ± 8.81	− 5.863	(− 13.531 to 1.805)	0.134
Superior	83.00 ± 15.84	89.55 ± 13.81	− 6.548	(− 18.886 to 5.789)	0.298
Nasal	74.88 ± 15.72	85.23 ± 13.84	− 10.351	(− 21.858 to 1.157)	0.078
Inferior	79.88 ± 17.29	89.97 ± 14.30	− 10.093	(− 22.277 to 2.091)	0.104
Temporal	77.38 ± 15.76	82.48 ± 11.18	− 5.109	(− 15.508 to 5.290)	0.336
**RNFL (μm)**					
Average	73.89 ± 15.93	85.57 ± 11.39	− 11.678	(− 23.336 to − 0.020)	0.050
Superior	90.56 ± 22.53	106.33 ± 13.61	− 15.778	(− 31.669 to 0.114)	0.052
Nasal	59.33 ± 12.86	69.27 ± 9.77	− 12.811	(− 32.126 to 6.504)	0.194
Inferior	95.56 ± 24.54	108.37 ± 17.17	− 9.933	(− 19.546 to − 0.321)	0.043
Temporal	50.89 ± 9.84	58.27 ± 14.57	− 7.378	(− 14.649 to − 0.107)	0.047
S/I ratio	0.96 ± 0.10	1.00 ± 0.14	− 0.039	(− 0.122 to 0.044)	0.356
S/T ratio	1.79 ± 0.34	1.90 ± 0.37	− 0.109	(− 0.365 to 0.147)	0.403
N/I ratio	0.63 ± 0.08	0.65 ± 0.08	− 0.013	(− 0.086 to 0.059)	0.715
N/T ratio	1.17 ± 0.18	1.23 ± 0.26	− 0.068	(− 0.218 to 0.082)	0.374

*β* Regression coefficient, *CI* Confidence interval, *DCP* Deep capillary plexus, *GCC* Ganglion cell complex,* I* Inferior, *MS* Multiple sclerosis, *N* Nasal, *NMOSD* Neuromyelitis optica spectrum disorder, *ON* Optic neuritis, *RNFL* Retinal nerve fiber layer, *RPC* Radial peripapillary capillaries, *S* Superior, *SCP* Superficial capillary plexus, *SD* Standard deviation, *T* Temporal.

### Comparison of SD-OCT and OCTA measurements in non-ON eyes

In MS-ON eyes, the RPC vessel density was reduced in the temporal quadrant (*p* = 0.028) compared with NMOSD-ON (Table 3, Fig. 1). The S/T and N/T ratios were lower in NMOSD eyes (*p* = 0.015 and *p* = 0.008, respectively). The vessel density of SCP and DCP, along with the GCC and RNFL thickness, were not significantly different between NMOSD-ON and MS-ON eyes (Table 3).

**Table 3:** Comparison of non-ON eyes between NMOSD and MS patients.

	NMOSD-ON	MS-ON	NMOSD-ON versus MS-ON		
SCP (%)	Mean ± SD	Mean ± SD	β	95% CI	*p* value
Average	48.29 ± 3.31	46.77 ± 2.80	1.511	(− 1.132 to 4.153)	0.263
Superior	49.31 ± 2.89	47. 53 ± 2.93	1.783	(− 0.672 to 4.238)	0.155
Nasal	47.26 ± 3.05	46.11 ± 3.36	1.147	(− 1.280 to 3.574)	0.354
Inferior	49.54 ± 4.14	48.11 ± 3.20	1.438	(− 1.676 to 4.552)	0.365
Temporal	47.08 ± 3.69	45.40 ± 2.79	1.678	(− 1.199 to 4.554)	0.253
**DCP (%)**					
Average	55.46 ± 1.50	55.89 ± 2.19	− 0.436	(− 1.637 to 0.765)	0.477
Superior	55.59 ± 1.50	55.92 ± 2.68	− 0.326	(− 1.608 to 0.955)	0.618
Nasal	55.48 ± 1.81	56.03 ± 2.06	− 0.553	(− 1.776 to 0.670)	0.376
Inferior	55.29 ± 1.87	55.88 ± 2.41	− 0.589	(− 1.785 to 0.606)	0.334
Temporal	55.48 ± 1.69	55.75 ± 2.31	− 0.268	(− 1.657 to 1.121)	0.705
**RPC (%)**					
Average	50.60 ± 1.71	49.87 ± 3.10	0.731	(− 0.750 to 2.213)	0.333
Superior	51.44 ± 1.95	51.67 ± 4.09	− 0.229	(− 2.084 to 1.626)	0.809
Nasal	46.66 ± 2.70	47.40 ± 3.82	− 0.742	(− 3.053 to 1.569)	0.529
Inferior	53.70 ± 2.09	52.56 ± 3.55	1.139	(− 0.580 to 2.858)	0.194
Temporal	51.94 ± 3.77	49.13 ± 4.08	2.810	(0.300 to 5.320)	0.028
S/T ratio	0.99 ± 0.07	1.06 ± 0.12	− 0.063	(− 0.114 to − 0.012)	0.015
N/T ratio	0.90 ± 0.07	0.97 ± 0.09	− 0.068	(− 0.118 to − 0.018)	0.008
I/T ratio	1.04 ± 0.08	1.07 ± 0.09	− 0.037	(− 0.094 to 0.019)	0.196
**GCC (μm)**					
Average	94.33 ± 9.10	90.74 ± 7.81	3.594	(− 4.252 to 11.440)	0.369
Superior	109.11 ± 12.92	102.24 ± 12.07	6.872	(− 4.118 to 17.862)	0.220
Nasal	106.00 ± 10.55	97.63 ± 12.62	8.370	(− 1.099 to 17.838)	0.083
Inferior	110.22 ± 12.10	101.91 ± 11.51	8.309	(− 2.122 to 18.740)	0.118
Temporal	100.33 ± 11.03	94.43 ± 9.81	5.899	(− 3.473 to 15.270)	0.217
**RNFL (μm)**					
Average	99.73 ± 12.26	92.20 ± 10.48	7.527	(− 1.584 to 16.638)	0.105
Superior	122.82 ± 18.19	113.07 ± 14.72	9.752	(− 2.876 to 22.379)	0.130
Nasal	79.64 ± 13.51	74.73 ± 12.57	9.467	(− 2.290 to 21.223)	0.115
Inferior	125.00 ± 14.71	115.53 ± 13.22	4.903	(− 5.517 to 15.324)	0.356
Temporal	71.45 ± 9.84	64.98 ± 10.86	6.477	(− 0.875 to 13.829)	0.084

*β* Regression coefficient, *CI* Confidence interval, *DCP* Deep capillary plexus, *GCC* Ganglion cell complex,* I* Inferior, *MS* Multiple sclerosis, *N* Nasal, *NMOSD* Neuromyelitis optica spectrum disorder, *ON* Optic neuritis, *RNFL* Retinal nerve fiber layer, *RPC* Radial peripapillary capillaries, *S* Superior, *SCP* Superficial capillary plexus, *SD* Standard deviation, *T* Temporal.

### Diagnostic accuracy of SD-OCT and OCTA parameters

**Figure 2:**
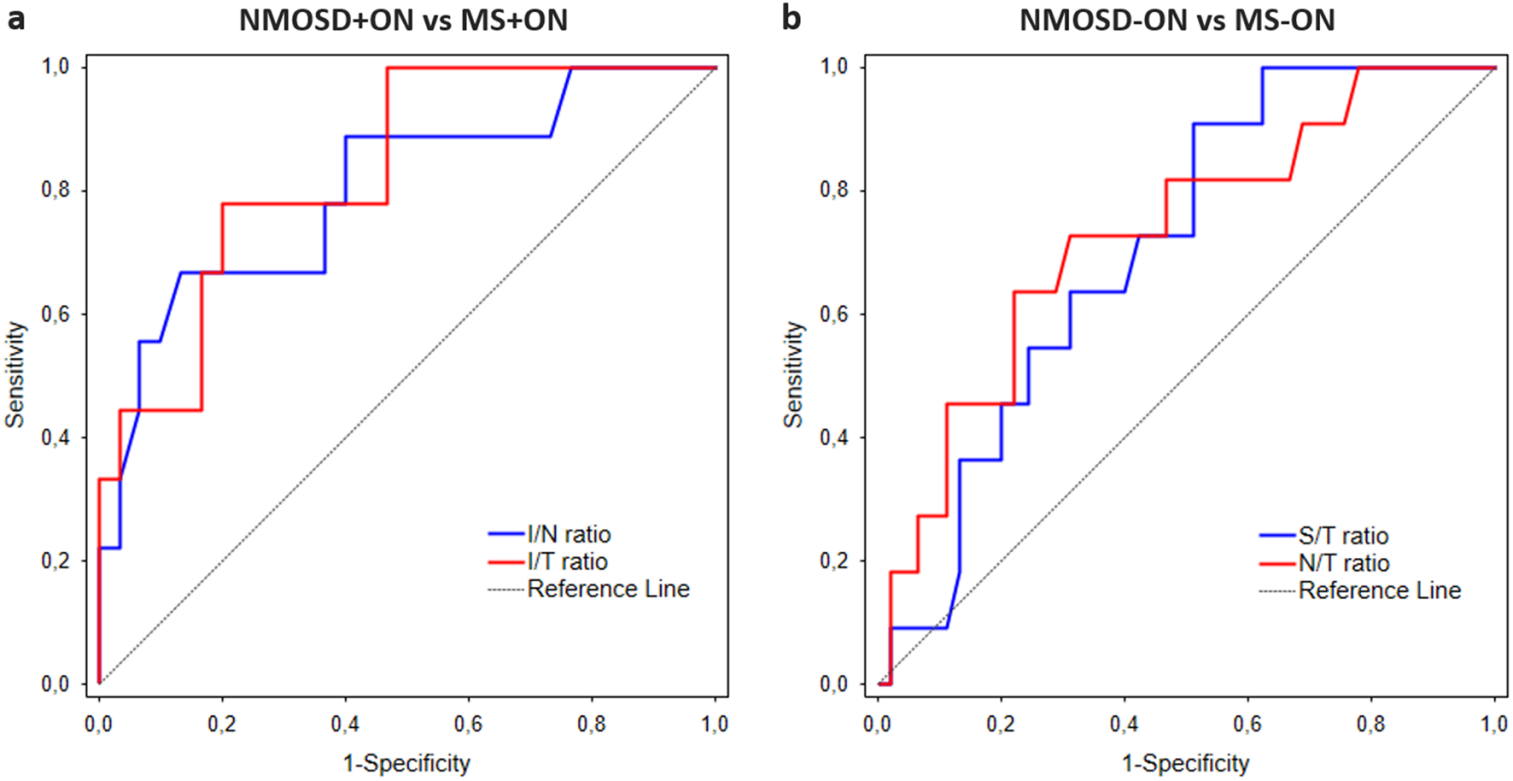
Receiver operating characteristic curves representing the best parameters differentiating NMOSD from MS for ON eyes (**a**) and non-ON eyes (**b**). *MS* Multiple sclerosis, *NMOSD* Neuromyelitis optica spectrum disorder, *ON* Optic neuritis.

The AUC values of the measurements and ratios that differed between the groups are presented in Table 4. The best parameters for differentiating NMOSD from MS eyes were I/N ratio (AUC, 0.802) and I/T ratio (AUC, 0.833) for ON eyes, and S/T ratio (AUC, 0.707) and N/T ratio (AUC, 0.728) for non-ON eyes (Table 4, Fig. 2a,b).

**Table 4:** Diagnostic accuracy of OCT angiography and spectral-domain OCT parameters.

	AUC	95% CI	*p* value	Cutoff value	Sensitivity (%)	Specificity (%)
**NMOSD + ON versus MS + ON**						
RPC (%)						
Superior	0.726	(0.536 to 0.916)	0.020	38.6	44.4	96.7
Inferior	0.780	(0.611 to 0.948)	0.001	52.7	100	43.3
S/T ratio	0.778	(0.605 to 0.950)	0.002	0.86	44.4	100
I/N ratio	0.802	(0.627 to 0.977)	0.001	1.04	66.7	86.7
I/T ratio	0.833	(0.692 to 0.975)	< 0.001	1.04	77.8	80.0
**RNFL (μm)**						
Temporal	0.606	(0.385 to 0.938)	0.348	64	100	26.7
Inferior	0.631	(0.404 to 0.859)	0.258	74	33.3	96.7
**NMOSD-ON versus MS-ON**						
RPC (%)						
Temporal	0.692	(0.515 to 0.869)	0.034	51.6	63.6	73.3
S/T ratio	0.707	(0.561 to 0.853)	0.006	1.06	90.9	48.9
N/T ratio	0.728	(0.563 to 0.894)	0.007	0.92	72.7	68.9

The optimal cutoff values were determined by Youden’s index.

*AUC* Area under the curve, *CI* Confidence interval, *I* Inferior, *MS* Multiple sclerosis, *N* Nasal, *NMOSD* Neuromyelitis optica spectrum disorder, *OCT* Optical coherence tomography, *ON* Optic neuritis, *RNFL* Retinal nerve fiber layer, *RPC* Radial peripapillary capillaries, *S* Superior, *T* Temporal.

## Discussion

This study demonstrated that the ON and non-ON eyes presented a different pattern of RPC vessel density and RNFL thickness reduction in NMOSD and MS patients. Additionally, the detection of disease-specific intra-eye alterations allowed us to create simple ratios to improve the differential accuracy between the diseases.

In ON eyes, the RPC vessel density was reduced in NMOSD eyes compared with MS eyes in the superior and inferior quadrants. As the large vessels leave the optic disc mainly at the superior and inferior borders, this sectoral predilection supports Green and Cree's observations that retinal vessels were attenuated in NMOSD eyes^11^. Although we did not observe similar vessel narrowing in NMOSD-ON, compared to MS-ON eyes, we can assume that the vascular alterations are related to ON and reflect the different immunopathogenic mechanisms of the diseases. In NMOSD, the AQP4-IgGs target the AQP4 water channels expressed on the retinal astrocytes and Müller cells. Their perivascular and end-foot processes, rich in AQP4, contribute to the formation and maintenance of the inner blood-retinal barrier by ensheathing the retinal capillaries^19,20^. It was shown that the blood vessels in NMO lesions were characterized by a narrow lumen and thickened, fibrotic or hyalinized walls^21,22^. Interestingly, we observed such changes in the peripapillary capillary plexus, but not in the macular microvasculature (both SCP and DCP), which did not differ between the NMOSD and MS patients.

Several studies independently investigated retinal vessel alterations in NMOSD and MS^23–28^. In NMOSD patients, Huang et al. and Chen et al. found lower peripapillary and parafoveal vessel density in ON and non-ON eyes than in control eyes^23,24^. Concerning MS, similar results were shown^25–28^. Recently, the first comparative analysis was presented. Lee et al. reported that the RPC vessel density was significantly reduced in all four sectors in NMOSD + ON eyes compared with MS + ON eyes^10^. Their findings were not consistent with ours, and the following aspects might influence the results. First, the cohort of NMOSD patients was smaller in our study. Second, different OCTA devices were used to obtain the measurements; thus, the diameter of the peripapillary annulus used to calculate the RPC vessel density was wider in our device protocol (1.00 mm; RTVue XR Avanti with AngioVue) than in the study of Lee et al. (0.75 mm; DRI OCT Triton Plus)^10^. Given uneven analyzed areas, the straightforward comparison of the results may be disputable. Third, they noticed that the results might not be generalizable to other ethnic groups. Moreover, they found that the vessel density in SCP was reduced in superior and inferior sectors in NMOSD eyes, but the possible reason for such capillary loss pattern was not further discussed^10^.

According to some studies, the GCC thickness differed between NMOSD and MS patients^9,10^, but the results of other studies did not confirm this^29,30^. In our ON and non-ON groups, the GCC thickness did not differ between NMOSD and MS. Regarding the RNFL thickness, the previous reports were also not consistent^7–10,29^. Our study found that the RNFL was significantly thinner in the inferior and temporal sectors in NMOSD + ON compared with MS + ON. Schneider et al. proposed using the N/T ratio to distinguish between NMOSD + ON and MS + ON eyes^9^. Thus, we tested the N/T ratio and the other three (S/I, S/T, and N/I) created based on the I and T sectors, but all of them did not differ significantly between the groups.

It was reported that the temporal axonal loss might be observed in MS-ON compared with NMOSD-ON eyes^29^. In our study, the RNFL thickness tended to decrease in this sector in MS-ON eyes, but the difference was not significant between the groups. However, we found that the RPC vessel density was reduced in the temporal quadrant in MS-ON compared with NMOSD-ON. Such a finding can be explained by neurodegeneration progress that continues during the course of MS independently from ON history and is more pronounced in those cases than in NMOSD^29^. As it was shown in the study by Spain et al., the reduction of the RPC vessel density accompanied the RNFL thickness loss in MS-ON eyes^25^.

In contrast to the insignificant RNFL-based ratios, the ratios based on the RPC vessel density differed significantly between NMOSD and MS, thus were further analyzed. For ON eyes, the I/T ratio and I/N ratio had the best AUC of 0.833 and 0.802, respectively, with the same cutoff value of 1.04 (Table 4). For non-ON eyes, S/T and N/T were the only ratios that showed differential diagnostic power (AUC of 0.707 and 0.728, respectively). Although the superior or inferior sectors of RPC vessel density could also be a possible differentiating parameter, the optimal cutoff value determined by Youden's index in our study might not be suitable for other cohorts. Therefore, we believed that the ratios based on the disease-specific intra-eye alterations would be more applicable.

Our study has several limitations. First, the group of NMOSD patients was small due to the low prevalence of this disease. Second, it was a single-center investigation. Therefore, further studies with larger diverse cohorts are required to confirm our observations. Finally, we included only AQP4-IgG seropositive NMOSD patients. Thus, our findings may not extend to seronegative NMOSD.

In conclusion, we demonstrated that a distinct pattern of RPC vessel density reduction in ON and non-ON eyes could facilitate the differentiation between NMOSD and MS. The use of OCTA-based simple ratios may improve the diagnostic accuracy.
